# Clinical Evaluation for Sublingual Immunotherapy With *Dermatophagoides farinae* in Polysensitized Allergic Asthma Patients

**DOI:** 10.3389/fmed.2021.645356

**Published:** 2021-08-05

**Authors:** Ai-zhi Zhang, Mei-e Liang, Xiao-xue Chen, Yan-fen Wang, Ke Ma, Zhi Lin, Kuan-kuan Xue, Li-ru Cao, Rong Yang, Huan-ping Zhang

**Affiliations:** ^1^Department of Pulmonary and Critical Care Medicine, Second Hospital Affiliated to Shanxi Medical University, Taiyuan, China; ^2^Department of Allergy Medicine, Shanxi Bethune Hospital, Shanxi Academy of Medical Sciences, Tongji Shanxi Hospital, Third Hospital of Shanxi Medical University, Taiyuan, China; ^3^Department of Pulmonary and Critical Care Medicine, First Hospital Affiliated to Shanxi Medical University, Taiyuan, China; ^4^Department of Internal Medicine, Shanxi Medical University, Taiyuan, China

**Keywords:** allergic asthma, sublingual immunotherapy, monosensitization, polysensitization, efficacy

## Abstract

**Background:** Many studies have demonstrated the efficacy of single-allergen sublingual immunotherapy (SLIT) in polysensitized patients with allergic rhinitis (AR), but less is reported in polysensitized patients with allergic asthma (AS).

**Method:** Data of 133 adult patients with house dust mite (HDM)-induced AS who had been treated for 3 years were collected. These patients were divided into the control group (treated with low to moderate dose of inhaled glucocorticoids and long-acting β2 agonists, *n* = 37) and the SLIT group (further treated with *Dermatophagoides farinae* drops, *n* = 96). The SLIT group contained three subgroups: the single-allergen group (only sensitized to HDM, *n* = 35), the 1- to 2-allergen group (HDM combined with one to two other allergens, *n* = 32), and the 3-or-more-allergen group (HDM combined with three or more other allergens, *n* = 29). The total asthma symptom score (TASS), total asthma medicine score (TAMS), and asthma control test (ACT) were assessed before treatment and at yearly visits. Forced expiratory volume in 1 s/forced vital capacity (FEV1/FVC) was assessed before treatment and at the end of SLIT.

**Results:** TASS and ACT scores in the control group were significantly higher than that in the single-allergen group and the 1- to 2-allergen group after 1, 2, and 3 years of SLIT and significantly higher than that in the 3-or-more-allergen group after 3-year SLIT (all *p* < 0.05). TAMS of the control group was significantly higher than that of the other three groups after 0.5, 1, 2, and 3 years of SLIT (all *p* < 0.05). FEV1/FVC in the control group was significantly higher than baseline after 3 years of immunotherapy (*p* < 0.05).

**Conclusion:** Patients sensitized to HDM with/without other allergens showed similar efficacy after 3 years of SLIT. However, the initial response of patients with three or more allergens was slower during immunotherapy process.

## Introduction

Asthma is a common chronic airway disease worldwide, affecting 18% of the populations in different countries (1). It has been recently proposed that asthma is a heterogeneous disease with different clinical phenotypes, and allergic asthma (AS) is one of the most important phenotypes, accounting for more than three-fifths of adult asthma (2). The World Allergy Organization Position Paper estimated that the global prevalence of allergic diseases was 10–40%, including 300 million patients with AS (3). In China, house dust mite (HDM) is the main allergen for patients with allergic diseases and the prevalence of sensitization was ~48% (4). HDM served as the main allergen in southern places while pollen might be the main allergen in the northern area in China. Kewu Huang and his colleagues reported in the *Lancet* that the prevalence of asthma in people aged over 20 was 4.2% in China, and the total number of patients had reached 45.7 million in 2019. However, 71.2% of 2,032 asthma patients had been never diagnosed by a physician in China and only 5.6% of them had received formal treatment (5).

Allergen immunotherapy (AIT) was born in 1,911 and has a history of more than 100 years. AIT is considered as the only option that may alter the natural course of allergic diseases (6). The latest international consensus on AIT has clearly stated that it has significant effect on allergic rhinitis (AR) and AS (7, 8). Sublingual immunotherapy (SLIT) and subcutaneous immunotherapy (SCIT) are the most common approaches for AIT. Considering the potential risks of AIT-associated adverse events (AEs), SLIT has been the preferred route of allergen administration compared to SCIT because of its better safety profile and the convenience of self-administration without medical supervision (9).

Polysensitization is a highly prevalent clinical phenomenon (10). Recent meta-analyses and systematic reviews showed that SLIT with a single allergen is efficacious in both monosensitized and polysensitized patients with AR (11, 12), while the effect of SLIT for AS is less complete. The purpose of this study was to evaluate the efficacy of single-allergen SLIT in polysensitized AS patients and provide an important reference for the specific immunotherapy in clinical practice.

## Methods

### Ethics Statement

The clinical trial was approved by the Medical Ethics Committee of Shanxi Medical University. All patients were informed of the trial details, and all patients signed written informed consent prior to performing any procedures.

### Study Design

This clinical trial was carried out in the Second Hospital Affiliated to Shanxi Medical University, Shanxi Bethune Hospital, Shanxi Academy of Medical Sciences, Tongji Shanxi Hospital, Third Hospital of Shanxi Medical University, and First Hospital Affiliated to Shanxi Medical University. Subjects aged 18–60 years were recruited from the outpatients that visited the departments between, March 15, 2016, and September 15, 2016. Patients' asthma symptoms and medication scores were recorded in our database; besides, suspected patients with bronchial asthma required the asthma control test (ACT). The number of samples collected is based on the actual number of patients who finished the treatment in each group.

Recruitment criteria included patients sensitized to aeroallergens aged 18–60 years, all of whom have been diagnosed with mild-to-moderate bronchial asthma; pulmonary function test FEV1 >70% of the predicted value; patients without previous AIT and with single allergen of HDM or HDM combined with other one to three or more allergens were recruited; other allergens are limited to inhaled allergens, but there is no limit to the types of allergens (including *Humulus scandens, Ragweed, Alternaria alternata, Cladosporium cladosporium, Aspergillus fumigatus*, cat hair, dog hairs, pillow material, Mulberry silk, and cockroach). The allergen protein homology between *Dermatophagoides farinae* (*D. farinae*) and *Dermatophagoides pteronyssinusanf* (*D. pteronyssinusanf*) is as high as 80% (13); therefore, both are included in the HDM allergen group. Sensitization to *D. farinae* and/or *D. pteronyssinusanf* and other inhaled allergens were confirmed by the presence of specific immunoglobulin E (sIgE) ≥ 0.70 KU/L (grade 2 and above) using the UniCAP system (Phadia, Uppsala, Sweden). Patients were excluded from the study if they had one of the following conditions: severe or uncontrolled asthma; uncontrolled or acute allergic diseases (anaphylactic shock); taking Angiotensin-Converting Enzyme (ACE) or β-blockers; serious psychological or mental illness; severe acute or chronic heart failure, kidney failure; pregnancy; and malignant tumors.

Demographic and clinical data were collected at each phase. According to the treatment method selected by the patients themselves rather than random grouping, eligible participants were divided into the control group (treated with low to moderate dose of inhaled glucocorticoid and long-acting β2 agonists) and SLIT group (treated with low to moderate dose of inhaled glucocorticoids and long-acting β2 agonists further treated with *D. farinae* drops); both the control group and the SLIT group were divided into single-HDM SLIT subgroup, HDM combining with other 1–2 allergen SLIT subgroup, and HDM combining with other 3 or more allergen SLIT subgroup, respectively. Patients were treated using standardized allergen *D. farinae* drops (Chanllergen; Zhejiang Wolwo Bio-Pharmaceutical Co., Ltd., Zhejiang, China) labeled from 1 to 5 containing proteins of 1, 10, 100, 333, and 1,000 μg/ml, respectively. The main components of vials 1–5 are the same, but the concentrations of protein are different. Information regarding normal drug dosage is shown in Table 1. The drug was self-administered daily at the same time and administered sublingually for 1–3 min before swallowing.

**Table 1:** SLIT drops dosing for patients.

**Week**	**Vial no**.	**Dose (drops)**						
		**1 d**	**2 d**	**3 d**	**4 d**	**5 d**	**6 d**	**7 d**
1	1	1	2	3	4	6	8	10
2	2	1	2	3	4	6	8	10
3	3	1	2	3	4	6	8	10
4–5	4	3	3	3	3	3	3	3
≥6	5	2	2	2	2	2	2	2

### Clinical Efficacy

During the treatment, patients were required to keep a diary recording of symptom and medication use. The investigators calculated the weekly average scores at every visit. The total asthma symptom score (TASS), total asthma medicine score (TAMS), ACT, and forced expiratory volume in 1 s/forced vital capacity (FEV1/FVC) were recorded. TASS was the sum of daytime asthma symptoms scores and nocturnal asthma symptoms scores. The daytime asthma symptoms were scored from 0 to 5 points according to the general severity of wheeze, shortness of breath, dyspnea, and cough and its impact on daily life. The nocturnal symptoms were scored from 0 to 4 points according to the frequency of nocturnal and early morning awakening by asthma (14). TAMS was calculated as follows (per day): one point for long-acting β2 agonists and two points for inhaled glucocorticoids; TAMS is the sum of all the recorded medicine scores (15). ACT is an effective tool to assess the degree of asthma control. Twenty-five points mean well-controlled, 20–24 points mean partially controlled, and it is uncontrolled when the points are below 20 (16). In addition, FEV1/FVC were measured to evaluate the pulmonary function of patients at the beginning and the end of immunotherapy by a pulmonary function tester.

### Safety Assessment

Safety profile was assessed according to AEs recorded in daily cards. All AEs were addressed under the instruction of the physicians.

### Patient Management

Initial clinical education and follow-up education were carried out for all the patients. The patient files were established to record symptoms, medication use, and AEs of patients at the beginning of the treatment. Telephone follow-ups were provided to patients to solve problems that occurred in the treatment process and to arrange the next follow-up visit.

### Statistical Analysis

Statistical analyses were performed using the SPSS 21.0 software (IBM Corp., Armonk, New York, USA). Data were assessed for normality and equal variation and results were expressed as mean ± standard deviation (SD). Kolmogorov–Smirnov test was performed to assess the normality of the distribution in continuous variables. Two-way analysis of variance (ANOVA) was used when the variables distributed normally. Otherwise, Kruskal–Wallis *H* test or Mann-Whitney *U*-test was performed. The two-tailed level of statistical significance was set at *p* = 0.05. Figures were plotted using GraphPad Prism 7.0 (Software Inc. La Jolla, CA, USA), and *p* < 0.05 was considered statistically significant.

## Results

### Subjects

A total of 230 participants [mean age, 41.19 ± 11.02 years; 33.91% female (*n* = 78), 66.09% male (*n* = 152)] were screened, of whom 133 completed the entire study in the control group (*n* = 37), single-allergen group (*n* = 35), 1- to 2-allergen group (*n* = 32), and 3-or-more-allergen group (*n* = 29). All groups were comparable with respect to gender, age, TASS, TAMS, and ACT, and there were no statistical differences in all items except the single-allergen group vs. the control group and the 1- to 2-allergen group in the FEV1/FVC% item (Table 2). The reasons for the patients' dropout included incomplete study (*n* = 20), withdrew consent (*n* = 9), lost to follow-up (*n* = 36), undetermined FEV1/FVC at the end of SLIT (*n* = 29), and others (*n* = 3).

**Table 2:** The demographic and clinical characteristics before treatment in the four groups.

**Character**	**Control group**	**Single-allergen group**	**1- to 2-allergen group**	**≥3 allergen group**	***p*-value**
Case No.	37	35	32	29	*p* > 0.05
Male	10	16	8	13	*p* > 0.05
Female	27	19	24	16	*p* > 0.05
Age (years)	42.16 ± 11.10	39.49 ± 11.60	41.88 ± 11.29	36.97 ± 10.25	All *p* > 0.05
TASS	5.35 ± 1.21	5.06 ± 1.37	4.78 ± 1.29	5.03 ± 1.35	All *p* > 0.05
TAMS	7.60 ± 1.01	7.86 ± 1.42	7.31 ± 1.89	7.55 ± 1.64	All *p* > 0.05
ACT	14.73 ± 2.24	15.37 ± 2.37	14.97 ± 2.44	14.00 ± 2.02	All *p* > 0.05
FEV1/FVC%	76.80 ± 5.54	73.65 ± 5.64	76.23 ± 4.95	75.13 ± 3.85	*p* < 0.05 (Single vs. Control/1–2)

*TASS, total asthma symptom score; TAMS, total asthma medicine score; ACT, asthma control test; FEV1/FVC%, forced expiratory volume in 1 s/forced vital capacity*.

### TASS Evaluation

**Figure 1:**
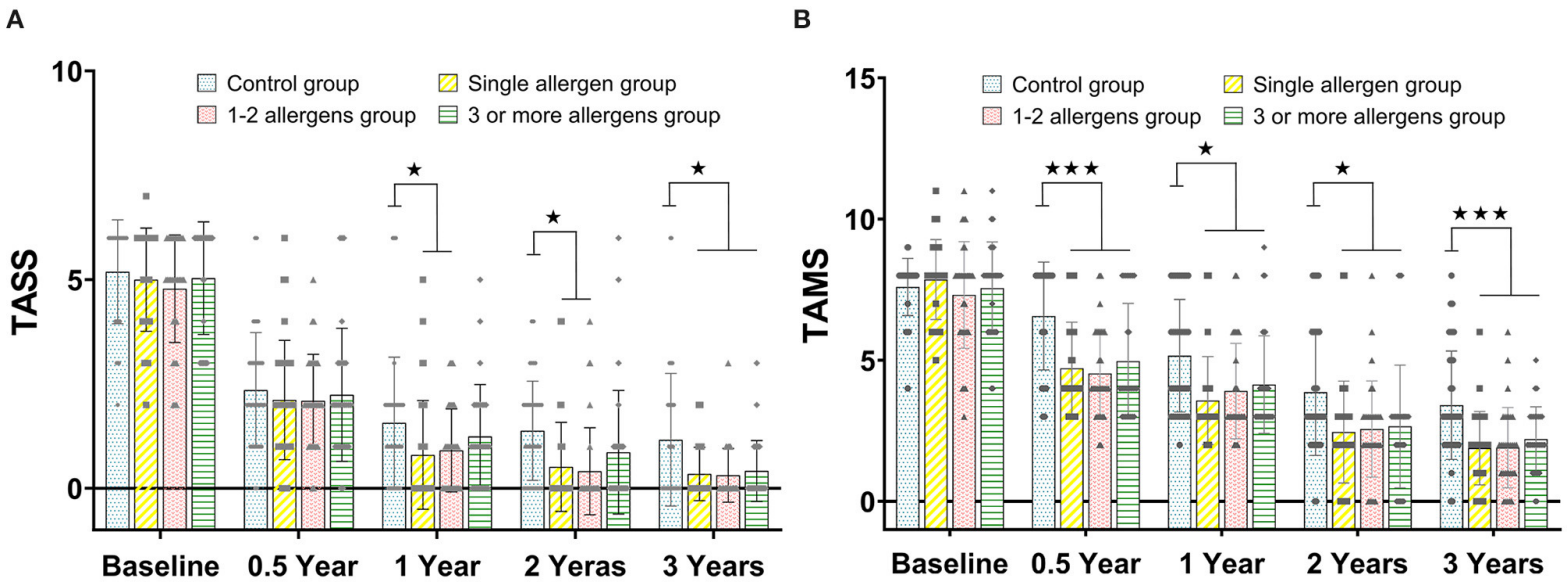
**(A)** The comparison of TASS scores between the control group, single-allergen group, 1- to 2-allergen group and 3-or-more-allergen group at different time points (mean ± SD). **(B)** The comparison of TAMS scores between the control group, single-allergen group, 1- to 2-allergen group and 3-or-more-allergen group at different time points (mean ± SD). ^⋆^*p* < 0.05, ^⋆⋆⋆^*p* < 0.001.

There was no statistical difference of TASS between the control group, single-allergen group, 1- to 2-allergen group, and 3-or-more-allergen group at baseline and 0.5 years (all *p* > 0.05), while there was a significant difference between the control group and the single-allergen group and 1- to 2-allergen group after immunotherapy after 1 and 2 years (all *p* < 0.05). The TASS score of the control group was significantly higher than that of the single-allergen group, the 1- to 2-allergen group, and the 3-or-more-allergen group at 3 years (*p* < 0.05, Figure 1A).

### TAMS Evaluation

As shown in Figure 1B, there was no statistical difference of TAMS between the control group, the single-allergen group, the 1- to 2-allergen group, and the 3-or-more-allergen group at baseline (all *p* > 0.05). However, TAMS in the control group was significantly higher than that in the single-allergen group, 1- to 2-allergen group and 3-or-more-allergen group after immunotherapy for 0.5 (*p* < 0.001), 1 (*p* < 0.05), 2 (*p* < 0.05), and 3 (*p* < 0.001) years.

### ACT Evaluation

**Figure 2:**
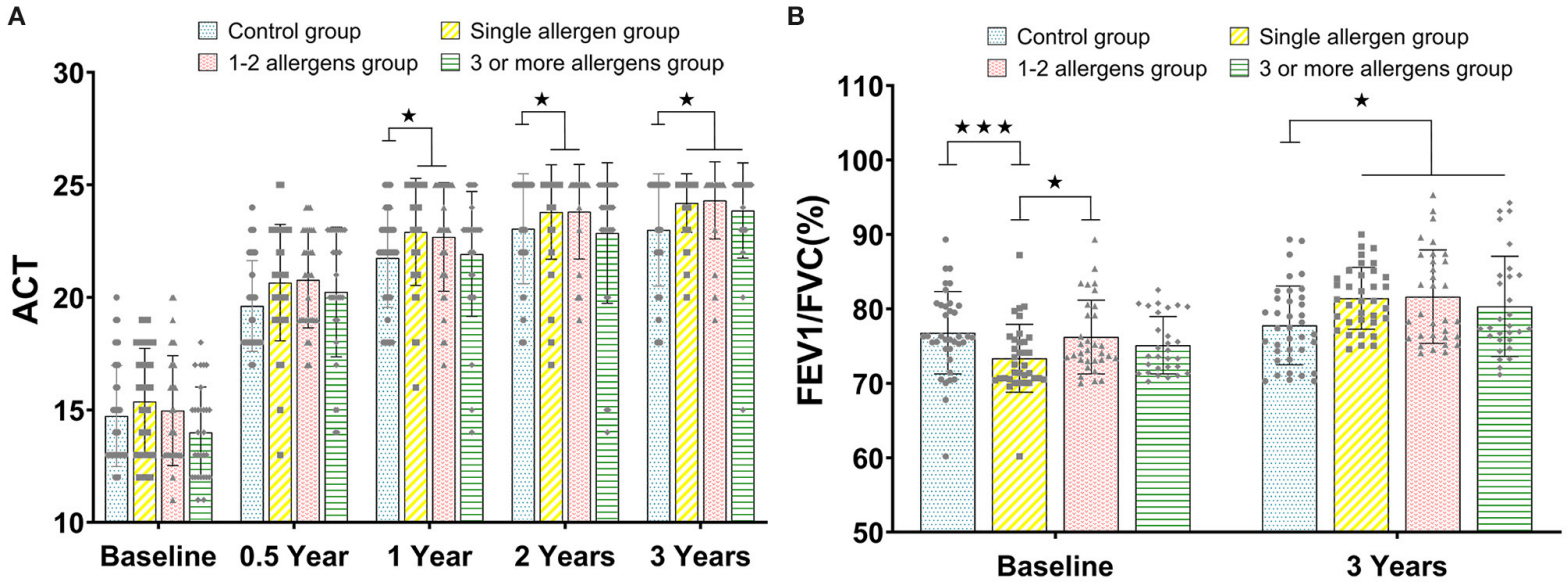
**(A)** The comparison of ACT between the control group, single-allergen group, 1- to 2-allergen group and 3-or-more-allergen group at different time points (mean ± SD). **(B)** The comparison of FEV1/FVC(%) between the control group, single-allergen group, 1- to 2-allergen group and 3-or-more-allergen group at different time points (mean ± SD). ^⋆^*p* < 0.05, ^⋆⋆⋆^*p* < 0.001.

Similar to the trend of TASS, there was no significant difference between all groups at baseline and 0.5 years (all *p* > 0.05). The values of ACT in the single-allergen group and 1- to 2-allergen group were significantly higher than that in the control group at 1 and 2 years (all *p* < 0.05), and the ACT score of the control group was significantly higher than that of the single-allergen group, 1- to 2-allergen group and 3-or-more-allergen group at 3 years (*p* < 0.05, Figure 2A).

### FEV1/FVC Evaluation

FEV1/FVC was directly assessed at baseline and after 3 years of immunotherapy. In the single-allergen group, the FEV1/FVC was significantly lower than the control group (*p* < 0.001) and 1- to 2-allergen group (*p* < 0.05) at baseline. Although there was no significant difference between the single-allergen group, 1- to 2-allergen group, and 3-or-more-allergen group (all *p* > 0.05) after immunotherapy for 3 years, there were significant differences between them and the control group (all *p* < 0.05, Figure 2B).

### Safety

No severe systemic adverse effects (AEs), anaphylaxis, asthma acute attack, or use of adrenaline were reported during the entire treatment period. Overall, 10 AEs occurred in the control group: 5 oral or sublingual itching, 3 swelling, and 2 diarrhea; 23 AEs occurred in the single-allergen group: 11 oral numbness or pruritus, 8 mild gastrointestinal reaction, and 4 aggravation of allergic symptoms; 9 numbness of tongue and 5 mouth ulcer make up all the AEs of the 1- to 2-allergen group, and there were 15 AEs in the 3-or-more-allergen group involving 7 oral numbness or pruritus and 8 mild gastrointestinal reaction. All AEs were relieved without any treatment within a week.

## Discussion

AS is one of the most common chronic diseases in all age population with high incidence and prevalence. As a disease-modifying therapy, SLIT is strongly recommended for treatment of AS patients, which has the potential to prevent the onset of new allergen sensitizations and the progression of respiratory allergies (7). Although SLIT has been widely studied, it mainly focuses on the area of single-allergen efficacy (17), objective indicators (18), and immune response pathway (19). Meanwhile, we found that most patients are not limited to a single allergen but multiple allergies in the actual clinical diagnosis and treatment process. What we are really concerned about is the efficacy of single-allergen SLIT for polysensitized patients. In this study, we selected patients allergic to *D. farinae* as the main body, as the research drug is specifically designed for HDM. In addition, other allergens are restricted to inhalation allergens, because inhalation allergens are more difficult to avoid in life. The number and types of allergens in each patient are also different in clinical treatment and the diagnosis and treatment ability of each hospital is different; therefore, this classification method is more conducive to guiding clinical practice for doctors.

In our study, there was a significant difference in FEV1/FVC between the single-allergen group, the control group, and the 1- to 2-allergen group, which indicated that FEV1/FVC in the single-allergen group is significantly lower than that in the other groups. The results of TASS and TAMS scores showed that efficacy of SLIT was consistent for patients in the single-allergen group and the 1- to 2-allergen group. Although patients in the 3-or-more-allergen group had slower onset of initial immunotherapy, they eventually achieved the same effect as the single-allergen group and the 1- to 2-allergen group. ACT identified as an effective tool for monitoring and assessing asthma (20). In present study, ACT was similar to the trend of TASS, although the response of patients in the 3-or-more-allergen group was slower than that in the single-allergen group and the 1- to 2-allergen group; the same effect as these two groups could be achieved in the end. The ACT results of patients were all above 20, and asthma symptoms were partially controlled after SLIT for 0.5, 1, 2, and 3 years. At baseline, FEV1/FVC in the single-allergen group was significantly lower than that in the control group and the 1- to 2-allergen group, which indicated that the lung function of patients in the single-allergen group was worse than that of patients in other groups. Additionally, the FVE1/FVC of patients in all three SLIT subgroups were significantly higher than that in the control group for the 3-year treatment, suggesting that lung function has been significantly improved whether patients have one or more allergens after 3-year SLIT.

However, there was no difference after treatment between the control group and some SLIT groups at certain time points, like the 0.5-year group of the TASS (Figure 1A) and ACT (Figure 2A). For this, we speculated that both the SLIT group and the control group were treated with symptomatic drugs at the early beginning. Therefore, the consistency in the symptoms of patients might be attributed to the role of inhaled glucocorticoids and long-acting β2 agonists in this process. After that, with the withdrawal of symptomatic drugs, the actual therapeutic effect has been shown.

The safety of SLIT has been demonstrated in multiple reviews of a large number of clinical trials (5, 21). No severe systemic AEs were reported in this trial. All the AEs were mainly local AEs such as transient oral itching and swelling. All the AEs were relieved within a week, with or without therapy. In addition, our study had relatively high compliance with 133/230 people completing the study; this was inseparable from the regular follow-up of medical staff. We also found that the total number of dropouts gradually increased as the follow-up time prolonged. Most patients quit SLIT mainly because their symptoms were under control. The period of dropout among these patients was mainly distributed during the follow-up period of 4–6 months. This might be related to the serious lack of knowledge of AS and SLIT treatment, the insufficient medical propaganda, and insufficient attention to asthma patients in China.

In this study, it was not easy for more than 100 patients to complete the treatment for 3 years; this was mainly due to our management of patients. We presented the information of patients at different time points; these data mainly came from our follow-up and collection of patients. The actual frequency of follow-up was much higher than the data we displayed. We believed that without our close contact with patients, these data could not be obtained. So, we look forward to sharing our follow-up methods with you in the future. However, the absence of a placebo control group was the main limitation in this study. Besides, we lacked the analysis of objective indicators of curative effect, and only the most important antibody component sIgE was partially introduced. Although there is no final conclusion on predictors, we expect to make our own voice in further research.

In conclusion, patients sensitized to HDM with/without other allergens showed similar efficacy after 3 years of SLIT. However, the initial response of patients with 3 or more allergens was slower during the immunotherapy process.

## Data Availability Statement

The raw data supporting the conclusions of this article will be made available by the authors, without undue reservation.

## Ethics Statement

The studies involving human participants were reviewed and approved by the raw data supporting the conclusions of this article will be made available by the authors, without undue reservation. The patients/participants provided their written informed consent to participate in this study.

## Author Contributions

A-zZ, X-xC, Y-fW, KM, K-kX, L-rC, and RY performed experiments, analyzed data, and reviewed the manuscript. M-eL organized the study and supervised experiments. H-pZ designed the project and prepared the manuscript. All authors contributed to the article and approved the submitted version.

## Conflict of Interest

The authors declare that the research was conducted in the absence of any commercial or financial relationships that could be construed as a potential conflict of interest.

## Publisher's Note

All claims expressed in this article are solely those of the authors and do not necessarily represent those of their affiliated organizations, or those of the publisher, the editors and the reviewers. Any product that may be evaluated in this article, or claim that may be made by its manufacturer, is not guaranteed or endorsed by the publisher.
